# Serum uric acid as a diagnostic biomarker for preeclampsia: comparison with renal indices and cerebroplacental ratio

**DOI:** 10.3389/fendo.2025.1761526

**Published:** 2026-01-06

**Authors:** Xiaoyan Chen, Kaimei Lian

**Affiliations:** 1 Shantou Chaonan Minsheng Hospital, Shantou, China; 2 First Affiliated Hospital of Shantou University Medical College, Shantou, China

**Keywords:** cerebroplacental ratio, diagnostic value, preeclampsia, renal function indices, serum uric acid

## Abstract

**Objective:**

To investigate the diagnostic performance of renal function-related indices (blood urea nitrogen (BUN), serum creatinine (sCr), serum uric acid (sUA), serum cystatin C (sCys C)) and the cerebroplacental ratio (CPR) for preeclampsia (PE).

**Methods:**

In a retrospective case-control design, we reviewed the medical records of 172 pregnant women (84 with PE and 88 normotensive controls) who delivered between 2021 and 2024. Independent risk factors were identified using logistic regression, and the Receiver Operating Characteristic (ROC) curve analysis was used to assess the discriminative ability of each parameter for detecting PE.

**Results:**

Compared with healthy controls, women with PE exhibited elevated sUA concentrations, greater pre-pregnancy body mass index (BMI), and lower CPR values (all *P* < 0.05). Multivariable analysis confirmed sUA as an independent predictor of PE, with a substantially increased odds ratio (OR) of 14.082 (*P* < 0.001). ROC curve analysis revealed that sUA had the highest area under the curve (AUC) for diagnosing PE (AUC = 0.837; 95% CI: 0.775-0.899), with a sensitivity of 75.00% and a specificity of 84.09%.

**Conclusion:**

Among the multiple renal function indices studied, sUA demonstrates the best diagnostic performance for PE and could serve as an effective biomarker for the clinical assessment of PE risk.

## 1 Introduction

Preeclampsia (PE) represents a major obstetric disorder unique to human pregnancy, contributing substantially to worldwide maternal and fetal complications and deaths (1–3). Although the exact pathogenic mechanisms remain incompletely understood, current evidence strongly suggests that inadequate placental invasion, generalised inflammatory activation, and impaired vascular endothelial function contribute to its development (4–6). Clinically, the diagnosis and severity assessment of PE primarily rely on blood pressure monitoring and the detection of proteinuria. However, these indicators have limitations in sensitivity and specificity, and some patients are diagnosed only after significant target-organ damage has occurred (7, 8).

In recent years, research has shown that PE patients often exhibit varying degrees of renal impairment. Serum indicators reflecting glomerular filtration and metabolic status—such as blood urea nitrogen (BUN), serum creatinine (sCr), serum uric acid (sUA), and serum cystatin C (sCys C)—may show characteristic changes. Among these, sUA has been identified as an independent risk factor for PE in numerous studies, owing to its pro-inflammatory and pro-oxidant properties, as well as its significant elevation during the pathophysiological process of PE (9–12). However, systematic comparative studies of its diagnostic performance relative to other renal function indices and ultrasound parameters, such as the cerebroplacental ratio (CPR), remain limited (6, 13). The CPR, a Doppler ultrasound index reflecting feto-placental hemodynamics and placental insufficiency, was included in this study to enable a multi-modal comparison with serum biomarkers, thereby enriching the clinical assessment perspective.

We hypothesised that among routinely available renal indices and CPR, sUA would demonstrate superior diagnostic performance for PE. Accordingly, this case-control study was designed to comprehensively assess renal function parameters and CPR measurements in pregnancies affected by PE, to determine their utility in supporting PE diagnosis and potentially facilitating earlier detection and clinical management.

## 2 Materials and methods

### 2.1 General information

This study adopted a retrospective case-control design. We enrolled 84 pregnant women with confirmed PE as the case group, alongside 88 randomly selected healthy pregnant women who served as the control group during the same period. To address potential confounding variables, participant groups were frequency-matched according to maternal age (within 3 years) and gestational week at delivery (within 1 week).

**Figure 1:**
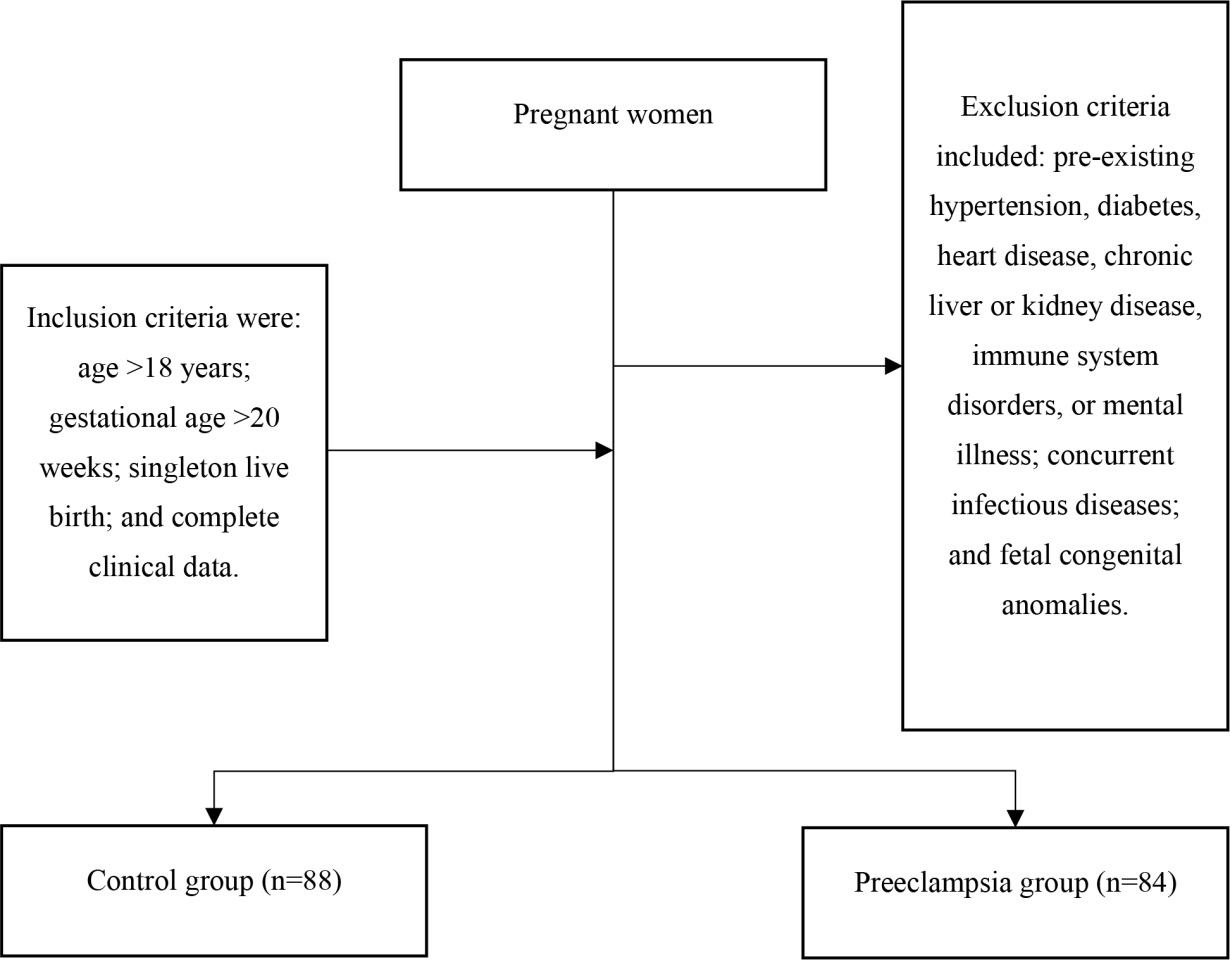
The flow chart of patient selection.

Inclusion criteria included: age > 18 years; gestational age > 20 weeks; singleton live birth; and complete clinical data. Exclusion criteria comprised: pre-existing hypertension, diabetes, heart disease, chronic liver or kidney disease, immune system disorders, or mental illness; concurrent infectious diseases; and fetal congenital anomalies (**Figure 1**).

PE was diagnosed based on the criteria established by the American College of Obstetricians and Gynaecologists (ACOG). The diagnostic criteria included new hypertension (systolic blood pressure ≥ 140 mmHg or diastolic blood pressure ≥ 90 mmHg) after 20 weeks of pregnancy, along with significant proteinuria (24-hour urine protein ≥ 0.3 g, protein-to-creatinine ratio ≥ 0.3, or random urine protein ≥ 1+). In the absence of proteinuria, PE was also diagnosed if any of the following criteria accompanied hypertension: platelet count <100,000/µL; serum creatinine > 1.1 mg/dL or a doubling of serum creatinine levels without other renal disease; elevated liver transaminases to twice the normal level; pulmonary edema; or a persistent headache with unexplained neurological symptoms (6–8, 14).

The Institutional Ethics Committee approved this retrospective investigation. Due to the retrospective nature of the research and the use of anonymised data, all participant identifiers were encrypted to ensure data confidentiality and security. In accordance with China’s “Ethical Review Measures for Biomedical Research Involving Humans,” this study met the criteria for a waiver of informed consent.

### 2.2 Study methods

We retrospectively collected clinical data for the enrolled pregnant women from our hospital’s Hospital Information System (HIS). The dataset included maternal characteristics like age, gravidity, parity, and body mass index (BMI). Prenatal Doppler parameters, specifically the pulsatility indices of the middle cerebral artery (MCA-PI) and umbilical artery (UA-PI), were documented for all study subjects within 7 days before delivery. The CPR was calculated as MCA-PI/UA-PI (6, 15, 16). Additionally, a 5 mL fasting venous blood sample was collected from each subject in the morning before delivery to measure serum levels of BUN, sCr, sUA, and sCys C using a Beckman automated biochemical analyzer and corresponding reagent kits (Beckman Coulter, USA). All laboratory analyses were performed in accordance with standard operating procedures and underwent internal quality control protocols (9).

### 2.3 Statistical analysis

We performed statistical analyses using SPSS (version 26.0) and MedCalc (version 18.0). Continuous variables that were normally distributed are presented as mean ± standard deviation and were analysed with Student’s *t*-test. Non-normally distributed data are presented as median with interquartile range and were tested using the Mann-Whitney *U* test. Categorical variables are reported as frequencies (percentages) and were evaluated using the Chi-square or Fisher’s exact tests. Multivariable logistic regression models were developed to examine independent relationships between each renal parameter and PE status.

Before constructing the models, we calculated variance inflation factors (VIF) for all renal indicators and confirmed the absence of substantial multicollinearity (all VIFs < 5). We developed four distinct regression models, each incorporating pre-pregnancy BMI and CPR as covariates along with one renal biomarker (BUN, sCr, sUA, or sCys C). The primary aim was to evaluate and compare the independent diagnostic contribution of each renal marker relative to PE, not to build a combined predictive model. Including all renal markers in one model could introduce multicollinearity (e.g., sCr and sCys C both reflect GFR), obscuring individual effects. This approach facilitates clinical interpretability, allowing clinicians to consider each marker separately in practice. Effect estimates are reported as odds ratios (ORs) accompanied by 95% confidence intervals (CIs). The diagnostic performance of each parameter for identifying PE was evaluated using receiver operating characteristic (ROC) analysis. We determined optimal cutoff values by maximising the Youden index. Comparisons of area under the curve (AUC) values among different indicators were performed using DeLong’s test. A two-sided p-value under 0.05 was deemed statistically significant. Given the retrospective design, *a priori* power calculation was not performed; however, *post-hoc* sensitivity analyses confirmed robust effect sizes for the primary comparisons.

## 3 Results

This study ultimately included 172 pregnant women, comprising 84 in the PE group and 88 in the healthy control group. The study groups demonstrated comparable baseline characteristics, with no significant differences in maternal age (*P* = 0.142), parity (*P* = 0.098), history of adverse gestational outcomes (*P* = 0.579), use of assisted reproductive technologies (*P* = 0.341), or incidence of pregnancy comorbidities, including gestational diabetes (*P* = 0.118), obstetric cholestasis (*P* = 0.237), and uterine fibroids (*P* = 0.267) (**Table 1**). However, significant intergroup differences were observed in several parameters: women with PE showed a higher pre-pregnancy BMI (*P* = 0.029) but lower CPR values (*P* < 0.001) compared to the control group. Furthermore, all evaluated renal function markers—BUN (*P* < 0.001), sCr (*P* < 0.001), sUA (*P* < 0.001), and sCys C (*P* = 0.001)—were significantly elevated in the PE cohort compared with healthy pregnant women (**Table 1**).

**Table 1:** Comparative analysis of demographic and renal functions between preeclampsia and control groups.

Variable	Control (n = 88)	Preeclampsia (n = 84)	*P*
Age (years)			
<35	69 (78.41%)	73 (86.90%)	0.142^a^
≥35	19 (21.59%)	11 (13.10%)	
Pre-gestational BMI (kg/m2), mean ± SD	22.02 ± 4.00	23.51 ± 4.83	0.029^c^
Primigravida			0.098^a^
No	60 (68.18%)	47 (55.95%)	
Yes	28 (31.82%)	37 (44.05%)	
History of adverse pregnancy			0.579^a^
No	56 (63.64%)	50 (59.52%)	
Yes	32 (36.36%)	34 (40.48%)	
IVF			0.341^a^
No	78 (88.64%)	78 (92.86%)	
Yes	10 (11.36%)	6 (7.14%)	
Diabetes during pregnancy			0.118^a^
No	69 (78.41%)	57 (67.86%)	
Yes	19 (21.59%)	27 (32.14%)	
Intrahepatic cholestasis during pregnancy			0.237^b^
No	88 (100.00%)	82 (97.62%)	
Yes	0	2 (2.38%)	
Uterine fibroids,			0.267^a^
No	76 (86.36%)	77 (91.67%)	
Yes	12 (13.64%)	7 (8.33%)	
Gestational weeks at measurement (weeks), mean ± SD	36.90 ± 2.09	36.60 ± 1.88	0.330^c^
CPR, mean ± SD	2.07 ± 0.48	1.75 ± 0.50	<0.001^c^
BUN (mmol/L), median (IQR)	3.34 (2.68, 3.95)	4.10 (3.51, 5.34)	<0.001^d^
sCr (μmol/L), median (IQR)	52.08 (48.75, 57.56)	60.62 (54.00, 71.00)	<0.001^d^
sUA (μmol/L), median (IQR)	334.06 (288.97, 391.77)	481.00 (409.50, 544.00)	<0.001^d^
sCys C (mg/L), median (IQR)	0.93 (0.86, 1.08)	1.05 (0.93, 1.24)	0.001^d^

SD, Standard deviation; IQR, Interquartile range; BMI, Body mass index; IVF, *In vitro* fertilisation; BUN, Blood urea nitrogen; sCr, Serum creatinine; sUA, Serum uric acid; sCys C, Serum cystatin C.

a, Determined with the Chi-square test; b, Determined with the Fisher’s exact test; c, Determined with the Student’s t-test; d, Determined with the Mann-Whitney U test.

To clarify the independent association of each indicator with preeclampsia, we constructed four multivariable logistic regression models. Multicollinearity diagnostics performed before model construction revealed that all VIFs for the included variables were below 5, indicating no severe multicollinearity. After adjusting for pre-pregnancy BMI and CPR in separate models, the results demonstrated that BUN (*P* < 0.001), sCr (*P* < 0.001), sUA (*P* < 0.001), and sCys C (*P* = 0.001) were all independent influencing factors for PE (**Table 2**). Among these, sUA exhibited the strongest association, with an OR of 14.082 (95% CI: 6.389-31.037). This finding suggests a very strong positive association between sUA and PE. However, the notably high odds ratio (OR = 14.082) for sUA warrants cautious interpretation. First, despite adjustment for pre-pregnancy BMI and CPR, residual confounding from unmeasured factors—such as dietary habits, medication use (e.g., antihypertensives), subclinical inflammation, or genetic predispositions—may have contributed to the magnitude of this association. Second, the cross-sectional design of our study limits causal inference; it is plausible that reverse causality plays a role, whereby PE-induced renal impairment, increased cellular turnover, and endothelial dysfunction elevate sUA levels, rather than sUA acting solely as a predictor of PE. Therefore, while our findings underscore a strong association between elevated sUA and PE, they should be interpreted within the constraints of the study design. Future longitudinal studies are needed to elucidate the temporal and causal relationships between these factors.

**Table 2:** Logistic regression analysis.

Variable	OR (95%CI)	*P*
Model 1		
BMI	4.070 (1.824 – 9.082)	0.001
CPR	3.382 (1.657 – 6.902)	0.001
BUN	7.549 (3.377 – 16.875)	<0.001
Model 2		
BMI	2.741 (1.320 – 5.692)	0.007
CPR	3.340 (1.628 – 6.854)	0.001
sCr	7.917 (3.586 – 17.477)	<0.001
Model 3		
BMI	2.292 (1.045 – 5.023)	0.038
CPR	2.863 (1.309 – 6.260)	0.008
sUA	14.082 (6.389 – 31.037)	<0.001
Model 4		
BMI	2.473 (1.247 – 4.904)	0.010
CPR	3.410 (1.732 – 6.712)	<0.001
sCys C	3.192 (1.618 – 6.295)	0.001

BMI, Body mass index; CPR, Cerebroplacental ratio; BUN, Blood urea nitrogen; sCr, Serum creatinine; sUA, Serum uric acid; sCys C, Serum cystatin C; CI, Confidence interval; OR, Odds ratio. VIF of BUN = 1.673. VIF of sCr = 1.799. VIF of sUA = 1.563. VIF of sCys C = 1.253.

**Figure 2:**
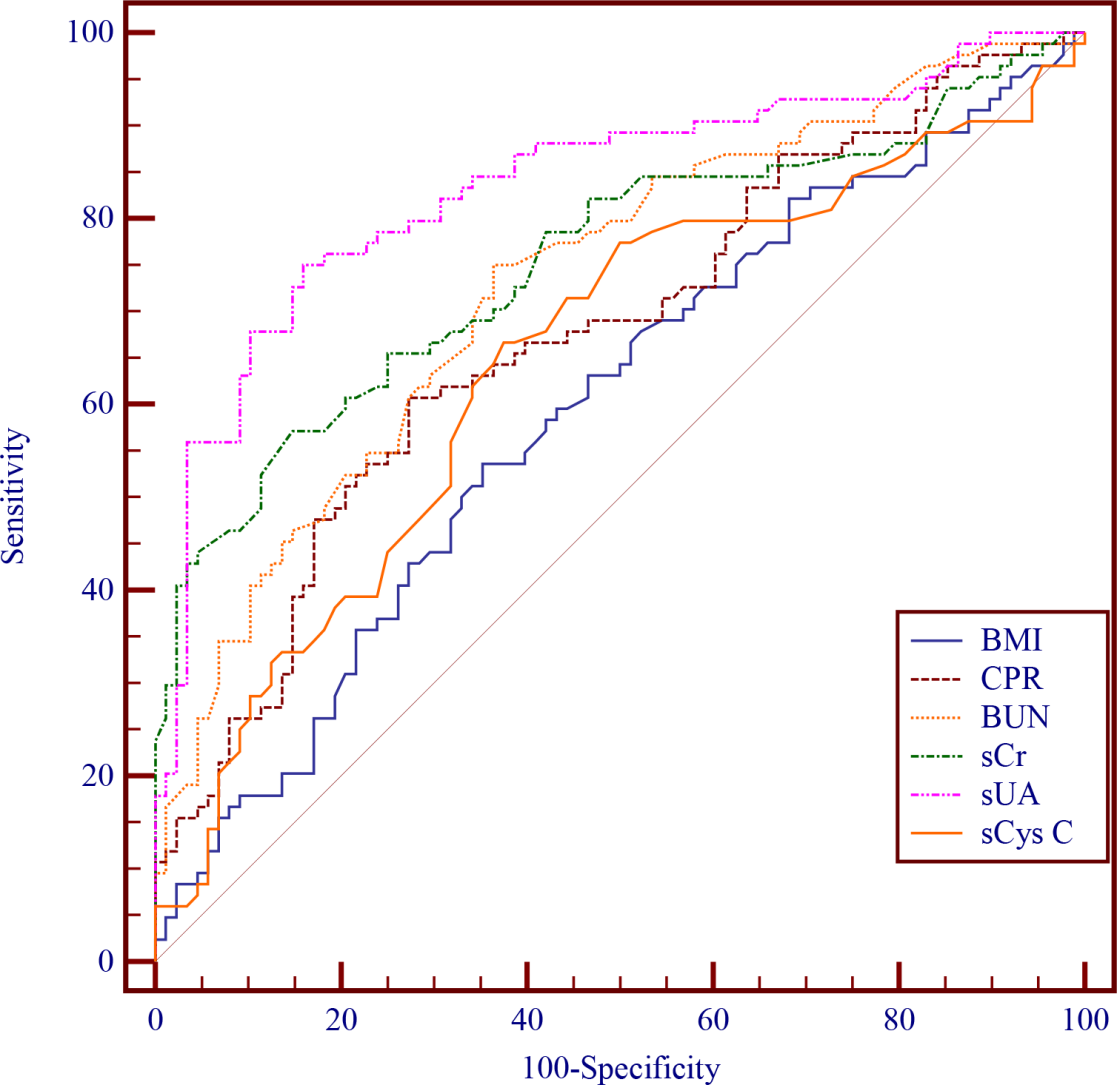
Diagnostic performances of BMI, CPR, BUN, sCr, sUA, and sCys C.

ROC curve analysis assessed the discriminative capacity of all investigated parameters for PE detection (**Figure 2**; participant enrollment details are provided in **Figure 1**). As summarised in **Table 3**, sUA emerged as the most effective single predictor, achieving an AUC of 0.837 (95% CI: 0.775-0.899). Through Youden index maximisation, the optimal diagnostic threshold was identified at > 408.79 μmol/L, corresponding to 75.00% sensitivity and 84.09% specificity. DeLong’s method showed that sUA’s AUC was significantly higher than those for pre-pregnancy BMI, CPR, and the remaining renal biomarkers (all *P* < 0.05). The cutoff of >408.79 μmol/L exceeds the typical upper limit for normal pregnancy (often 340-360 µmol/L in late pregnancy), supporting its clinical relevance.

**Table 3:** Diagnostic performances of BMI, CPR, BUN, sCr, sUA, and sCys C.

Data	Cutoff value	SEN (%)	SPE (%)	AUC (95%CI)
BMI	>22.7	53.57 (45/84)	64.77 (57/88)	0.595^cde^ (0.510 – 0.680)
CPR	≥1.8252	60.71 (51/84)	72.73 (64/88)	0.675^e^ (0.594 – 0.755)
BUN	>3.51 mmol/L	75.00 (63/84)	63.64 (56/88)	0.732^ae^ (0.657 – 0.807)
sCr	>59.47 μmol/L	57.14 (48/84)	85.23 (75/88)	0.754^aef^ (0.680 – 0.828)
sUA	>408.79 μmol/L	75.00 (63/84)	84.09 (74/88)	0.837^abcdf^ (0.775 – 0.899)
sCys C	>0.97 mg/L	66.67 (56/84)	62.50 (55/88)	0.646^de^ (0.563 – 0.730)

BMI, Body mass index; BUN, Blood urea nitrogen; sCr, Serum creatinine; sUA, Serum uric acid; sCys C, Serum cystatin C; CPR, Cerebroplacental Ratio; SEN, Sensitivity; SPE, Specificity; AUC, Area under the curve; CI, Confidence Intervals.

a = Compared with the BMI, *P*<0.05; b = Compared with the CPR, *P*<0.05; c = Compared with the BUN, *P*<0.05; d = Compared with the sCr, *P*<0.05; e = Compared with the sUA, *P*<0.05; f = Compared with the sCys C, *P*<0.05.

## 4 Discussion

This retrospective case-control study found that among multiple renal function indices and the CPR, sUA demonstrated the highest diagnostic performance for PE. sUA was significantly elevated in PE patients, remained an independent risk factor after multivariable adjustment, and showed the best diagnostic performance in ROC analysis (AUC = 0.837). We acknowledge that trimester-specific reference intervals were not used in this study, as all measurements were taken near the time of delivery. This is noted as a limitation, and we suggest that future studies establish gestational-age-specific cutoffs.

Our study confirms that sUA is a strong predictor of PE, a finding consistent with previous research (17–19). The notably high OR of 14.082 for sUA in our study, while requiring cautious interpretation within the retrospective design, strongly suggests a pivotal role in the pathophysiology of preeclampsia. It is noteworthy that our study found the diagnostic performance of sUA (AUC = 0.837) to be superior to that of sCr (AUC = 0.754) and sCysC (AUC = 0.646). This observation may be attributable to the fact that sCr and sCys C primarily reflect the glomerular filtration rate and exhibit relatively delayed changes during pregnancy (20). In contrast, sUA is not only influenced by renal function but also directly participates in the core pathological processes of PE, leading to earlier and more pronounced changes (17, 21).

The elevation of sUA in PE is not merely a passive byproduct of impaired renal function but rather reflects its active involvement as a key molecule in the disease process. A pivotal initiating factor in this cascade is placental ischemia and hypoxia, a hallmark of PE pathophysiology. These conditions lead to extensive cellular destruction and accelerated purine metabolism, thereby serving as a primary source for increased uric acid production (22). Significantly, uric acid subsequently functions as an endogenous damage-associated molecular pattern (DAMP) that triggers Nucleotide-binding Oligomerisation Domain-like Receptor Protein 3 (NLRP3) inflammasome activation. This activation, in turn, enhances the secretion of pro-inflammatory mediators, including Interleukin-1 beta (IL-1β), and amplifies systemic inflammatory processes (23). In parallel, uric acid impairs endothelial function by suppressing endothelial Nitric Oxide Synthase (eNOS) enzymatic activity, thereby diminishing nitric oxide generation and compromising its biological efficacy, ultimately promoting vascular endothelial pathology (24, 25).

Furthermore, a hyperuricemic state can induce oxidative stress in trophoblasts, thereby aggravating shallow placental implantation (26–28). Therefore, the sUA level can be considered a crucial hub linking the initial placental dysfunction (ischemia) to subsequent systemic inflammation and vascular endothelial injury.

The findings of this study carry clear clinical significance. sUA is an economical and readily accessible test within routine prenatal care. Its incorporation into the PE risk assessment framework could serve as a valuable adjunct to blood pressure and proteinuria measurements, potentially aiding in identifying early-stage patients who exhibit subtle blood pressure elevation but already harbour underlying pathophysiological alterations. Although sUA alone is not sufficient to replace the current diagnostic criteria, its high specificity (84.09%) indicates that a significantly elevated sUA level (> 408.79 μmol/L) should prompt clinicians to maintain a high index of suspicion for PE. Consequently, clinicians can facilitate earlier initiation of intensified monitoring and intervention based on this finding, potentially improving maternal and neonatal outcomes.

Several limitations should be considered when interpreting our findings. The retrospective design from a single institution introduces potential selection bias, and the relatively small cohort size may limit statistical power. Additionally, although we established the diagnostic utility of sUA, our analysis did not comprehensively examine its correlations with specific maternal-fetal complications, including fetal growth restriction or premature delivery. Gestational diabetes was present in some participants, but there was no significant difference between groups (**Table 1**). Baseline renal function variations and medication use (e.g., antihypertensives) were not systematically recorded in our retrospective data. Dietary and lifestyle factors that influence uric acid levels were not assessed. We state that these unmeasured factors may represent residual confounding and suggest that future prospective studies should systematically collect such data to address this issue.

Furthermore, the study did not include other emerging biomarkers (e.g., the soluble fms-like tyrosine kinase-1 to placental growth factor ratio (sFlt−1/PlGF)) for parallel comparison. While this study focused on routinely available indices, future research directly comparing the diagnostic and prognostic performance of sUA against emerging biomarkers—such as sFlt-1/PlGF, PlGF itself, or lactate dehydrogenase (LDH)—would be valuable to delineate further its relative clinical utility and potential role within multi-marker panels. Future research could build upon this work by developing a multi-parameter predictive model that incorporates sUA and further validating its clinical utility through prospective, multicenter, large-scale studies. The potential of sUA to guide clinical interventions warrants further exploration.

## 5 Conclusion

In summary, this study demonstrates that, among pre-pregnancy BMI, CPR, and multiple renal function indices, sUA possesses the best diagnostic performance for PE. As an economical, readily available test, sUA shows promise as a clinical adjunct to standard blood pressure and proteinuria measurements. However, prospective validation is needed before widespread clinical adoption can be recommended.

## Data Availability

The raw data supporting the conclusions of this article will be made available by the authors, without undue reservation.
